# Arecoline aggravates acute ulcerative colitis in mice by affecting intestinal microbiota and serum metabolites

**DOI:** 10.3389/fimmu.2023.1197922

**Published:** 2023-07-10

**Authors:** Hu Zhao, Taohui Ding, Yanglin Chen, Wenbin Yang, Jun Rao, Dan Liu, Bo Yi

**Affiliations:** ^1^ School of Pharmacy, Nanchang University, Nanchang, China; ^2^ 2nd Abdominal Surgery Department, Jiangxi Cancer Hospital, Nanchang, China

**Keywords:** arecoline, ulcerative colitis, gut microbiota, metabolomics, inflammatory responses

## Abstract

Arecoline is an alkaloid extracted from betel nut, which has various pharmacological effects. In the present study, we showed that arecoline aggravated experimental acute ulcerative colitis (UC) induced by dextran sodium sulfate (DSS) in mice. We measured body weight and colon length, evaluated disease activity index, colon pathology sections, and levels of colonic inflammatory factors. Arecoline exacerbated the clinical signs of UC and the colonic inflammatory response in mice. The results of 16S rRNA sequencing of fecal samples showed a significant decrease in the percentage of probiotic bacteria *Ligilactobacillus*, *Limosilactobacillus* and *Lactobacillus* and a significant increase in the percentage of conditionally pathogenic bacteria *Odoribacter* and *Bacteroides* after arecoline treatment. Serum untargeted metabolomics showed that arecoline intervention reduced the levels of ergothioneine, pentostatin, diadenosine tetraphosphate and other metabolites and modulated nicotinate and nicotinamide metabolism, metabolic pathways, glyoxylate and dicarboxylate metabolism, and other metabolic pathways of intestinal microorganisms. According to the combined microbial and metabolite analysis, arecoline influences metabolite levels by modulating the intestinal microbiota. In summary, it was found that arecoline treatment exacerbated colonic injury and intestinal inflammatory responses in UC mice, disrupted the host’s intestinal flora, and affected changes in flora metabolites, thereby exacerbating the development of colonic inflammation. Therefore, the consumption of betel nut can be associated with the risk of aggravating UC.

## Introduction

Ulcerative colitis (UC) is a form of inflammatory bowel disease (IBD) that has become a globally widespread and disease that poses a serious health risk given its increasing incidence (1). UC is a chronic idiopathic inflammatory disease with common symptoms including blood in the stool and diarrhea and mainly involves the colon and rectum. Its pathogenesis includes environmental factors, an unhealthy lifestyle, immune imbalance and dysbiosis of the gut microbiota (2, 3).Meanwhile, UC is also closely related to colorectal cancer, and studies have shown that long-term UC increases the risk of developing colorectal cancer (4).

Gut microbiota are closely related to the development of UC, and dysbiosis of the gut flora can increase the severity of UC (5). Studies have shown that a significant increase in the abundance of *Bacteroidota* and a relative decrease in the abundance of *Firmicutes* in the intestines of UC patients and reduced ratio of *Firmicutes* to *Bacteroides* (F/B) contribute to the development of UC (6).5-aminosalicylate sodium (5-ASA), cyclosporine, and glucocorticoids are the traditional drugs used to treat UC. However, these drugs are associated with serious side effects. Probiotics have fewer side effects than traditional drugs and can treat and alleviate the occurrence of UC (7). The probiotic *Lactobacillus plantarum* HNU082 (Lp082) was found to protect the mucosal barrier of the intestine, modulate the gut microbiota and reduce the symptoms of intestinal inflammation for the purpose of treating UC (8). Furthermore, live and pasteurized *Akkermansia muciniphila* effectively alleviated the symptoms of colitis in mice by promoting the proliferation of beneficial intestinal bacteria, increasing the production of short-chain fatty acids and suppressing intestinal inflammation (9).

Herbal extracts have also been extensively studied for their ability to inhibit the development of UC in mice. For example, the polyphenol extract of *Thymus vulgaris L*. could protect the intestinal epithelial barrier, modulate the intestinal microbiota, and inhibit the TLR4/NF-κB-NLRP3 inflammatory vesicle pathway to improve UC in mice (10). Intestinal flora metabolites are closely related to the maintenance of the intestinal barrier and the balance of the intestinal immune microenvironment. Short-chain fatty acids (SCFAs) have been shown to have immunomodulatory effects by reducing the production of pro-inflammatory factors to reduce the inflammatory response and mitigate the development of UC (11, 12).

Arecoline is the active ingredient of betel nut, and its pharmacological action has important effects on the nervous, cardiovascular, endocrine, and digestive systems (13–15). Some studies have shown that arecoline can increase inflammatory cell infiltration in the oral epithelium and affect the synthesis of various inflammatory mediators, thus leading to the development of oral epithelial fibrosis (16, 17). More importantly, long-term stimulation by arecoline may lead to further development of oral squamous cell carcinoma (18, 19). One study found that consumption of betel nut induces the peripheral blood mononuclear cells (PBMC) to secrete inflammatory cytokines such as IL-1β, IL-8, TNF-α, and IL-6, leading to their increased circulation (20). In long-term studies, arecoline has great research significance for its role in the body (21). However, it remains unexplored whether the development of UC is affected by arecoline.

In this study, the effect of arecoline on dextran sodium sulfate (DSS)-induced UC was investigated. We assessed the clinical signs by observing the colonic length, weight change, and disease activity index of mice, and the inflammatory response based on their colonic pathological changes and the concentrations of colonic tissue inflammatory factors. We also performed 16S rRNA sequencing on fecal samples and untargeted metabolomics analysis on serum samples to explore the specific mechanism of arecoline action on UC. The results suggest that arecoline may exacerbate DSS-induced UC by affecting the intestinal microbiota and its metabolites.

## Materials and methods

### Animal experimental design

We purchased 18 C57BL/6 male mice (age: 6–8 weeks) from Jiangsu Jicuiyaokang Biotechnology Co (Nanjing, China). The mice were housed at the Jiangxi Academy of Sciences, Royo Biotech Co., Ltd. (Nanchang, China). They were housed at a temperature of 25°C with a 24-h light-dark cycle. Mice had *ad libitum* access to food and water. After 7 days of acclimation, the mice were divided into three groups (n=6 per group): (1) Control group that was allowed *ad libitum* access to food and water; (2) DSS group: *ad libitum* diet was allowed for 1–7 days and 3% DSS (Meilunbio, Dalian) was added to the drinking water for 8–14 days; (3) Arec group: ad libitum diet was allowed for 1–7 days, 3% DSS was added to the drinking water for 8–14 days, and 5 mg/kg arecoline (Yuanye, Shanghai) was administered daily by tube feeding.

### Colonic length and histological analysis

Mice were killed after anesthesia, and their colon was dissected out and its length measured. Colon samples were preserved and fixed in 4% paraformaldehyde solution and then embedded in paraffin. The colon tissue was cut into 4-µm–thick sections, and then the colon sections were stained using hematoxylin and eosin (H&E). The stained slides were examined under an inverted microscope and photographed.

### Assessment of disease activity index

Throughout the experiment, the mice were weighed daily and fecal conditions were recorded. The stool properties and body weight were scored together, and the scores were summed and recorded as the DAI (22). Assessment was performed according to the following scoring criteria:

*weight change*: 0, no weight loss; 1, weight loss of 1–5%; 2, weight loss of 5–10%; 3, weight loss of 10–15%; 4: weight loss of >15%.
*stool consistency*: 0, normal; 2, dilute stool; 4, diarrhea.
*blood in the stool*: 0, no blood; 2, visual pellet bleeding; 4, severe bloody stool and blood around the anus.

### Enzyme-linked immunosorbent assay

Colon tissue stored at -80°C was thawed and mixed with phosphate-buffered saline (PBS) at a volume of 1:9 by weight and placed in a tissue grinder for homogenization, followed by centrifugation at 3000×g for 4 min at 5°C. We quantified the supernatant after centrifugation for quantitative ELISA (Shenkebio, Wuhan) of TNF-α, IL-1β, and IL-6 according to the manufacturer’s instructions.

### 16S rRNA sequencing

Mice fecal samples were collected after excretion and stored immediately in liquid nitrogen. We extracted genomic DNA by the Cetyltrimethylammonium Bromide (CTAB) method. The purity and concentration of DNA was assessed using 2% agarose gel electrophoresis; the DNA samples were diluted in sterile water to 1 ng/μl. After electrophoretic detection, the DNA was purified using magnetic beads and quantified by enzyme marker. Electrophoretic detection was again performed using 2% agarose gels, followed by recovery of target bands using Qiagen’s gel recovery kit (Qiagen, Germany). We used TruSeq^®^ DNA PCR-Free sample preparation kit to construct libraries. Libraries were constructed and quantified by Qubit and Q-PCR, and the qualified libraries were sequenced using NovaSeq6000. 16S rRNA sequencing was provided by Metware Biotechnology Co., Ltd. (Wuhan, China).

The data was efficiently processed by FLASH (v1.2.11) and Qiime (v1.9.1). The valid data were clustered using the Uparse algorithm (USEARCH v7) to assign sequences with a similarity ≥97% to the same operational taxonomic units (OTUs). Taxonomic information was annotated using the Mothur algorithm of the SILVA v138.1 (http://www.arb-silva.de/) database. The Shannon index, Simpson index, ACE index, and Chao1 index were calculated using R software (v4.1.2) and QIIME (v1.9.1) to assess alpha diversity. We also performed principal co-ordinate analysis (PCoA) and analysis of similarities (ANOSIM) to assess beta diversity using QIIME and R software. Last, LEfSe analysis using LEfSe software was used to screen for LDA score ≥4.

### Untargeted metabolomics

After thawing and vortexing the samples, 50 μL sample and 300 μL extract containing the internal standard (acetonitrile:methanol=1:4, V/V) were added to the centrifuge tube. The sample was again vortexed for 3 min and centrifuged at 12,000 rpm for 10 min at 4°C. Then, the 200 μL supernatant was collected and placed at -20°C for 30 min, followed by re-centrifuging at 12,000 rpm for 3 min at 4°C. This supernatant was collected for LC-MS analysis (Metware Biotechnology Co., Ltd., Wuhan, China). Samples were collected and analyzed according to the LC-MS system machine instructions. Meanwhile, we annotated the identified metabolites using the Kyoto Encyclopedia of Genes and Genomes (KEGG) compound database (http://www.kegg.jp/kegg/compound/) and then mapped the annotated metabolites to the KEGG pathway database (http://www.kegg.jp/kegg/pathway.html). Orthogonal partial least squares discriminant analysis of the three groups of differential serum metabolites was performed by R software. VIP (VIP>1) and P-value (P<0.05, Student’s *t*-test) were used for differential metabolite screening in both groups. For multiple group analysis, VIP (VIP>1) and P-value (P-value<0.05, ANOVA) were used for differential metabolite screening.

### Statistical analysis

Data are expressed as arithmetic mean ± standard error of the mean (SEM). Data were analyzed and counted using GraphPad Prism 8.0. Statistically significant differences between the groups were assessed by one-way analysis of variance (ANOVA). Bivariate correlations were calculated using Pearson’s r coefficient. Heat maps were constructed using R software (v4.1.2). We investigated the correlation between gut microbiota and serum metabolites using Spearman’s correlation analysis. P<0.05 was considered to indicate statistically significant differences.

## Results

### Effect of arecoline on clinical symptoms of colitis in mice

**Figure 1:**
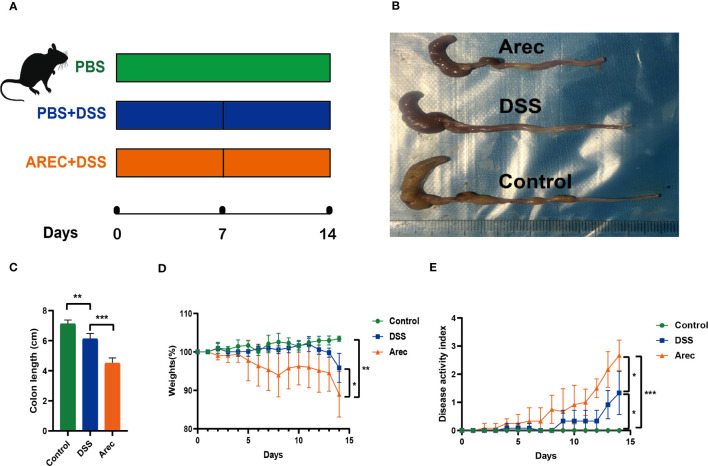
The effect of arecoline on clinical symptoms in mice with DSS-induced UC. **(A)** Establishment of a mouse UC model and treatment of arecoline. **(B)** Representative images of the colon in the Arec, DSS, and control groups of mice. **(C)** Colonic length in mice. **(D)** Body weight changes of the three groups of mice during the experiment. **(E)** Disease activity index of the three groups of mice. P<0.05 was statistically significant. *P<0.05, **P<0.01, and ***P<0.001.

We used 3% DSS solution to induce colitis in mice to establish the UC model, which was used to study the effect of arecoline on colitis. Details of the study groups are presented in **Figure 1A**. By measuring the colon length of the three groups of mice, we found that mice in the DSS group had a shorter colon length than those in the control group (P<0.01); however, the shortening of the colon was more severe in the Arec group (P<0.001) (**Figures 1B, C**). With respect to weight loss, there was a significant difference in weight between the arecoline and control groups at day 14 (P<0.01); furthermore, arecoline exacerbated the weight loss in the DSS group mice (**Figure 1D**). We used the DAI scores to analyze body weight change and fecal properties (blood in stool and stool consistency) in all mice, and arecoline aggravated the clinical signs of DSS-induced acute ulcerative colitis in mice (**Figure 1E**).

### Effect of arecoline on the inflammatory response in mice colon

**Figure 2:**
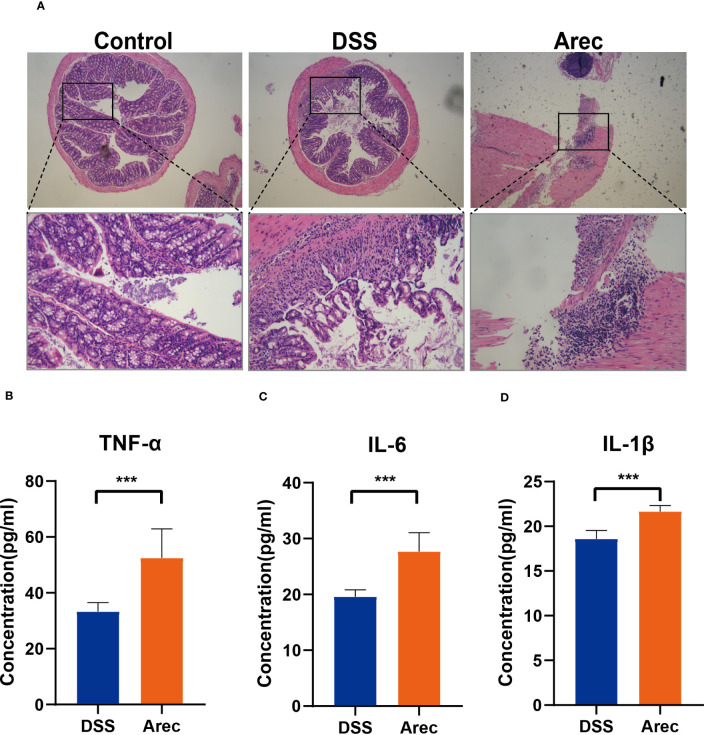
Histopathological sections of mouse colon with changes in inflammatory factors. **(A)** H&E stained images of mouse colon tissue (magnification 40×, 200×). **(B–D)** The concentration of inflammatory factors including TNF-α, IL-6, and IL-1β in the colonic tissue of mice in the DSS and Arec groups. P<0.05 was statistically significant. ***P<0.001.

We observed by HE staining of colonic sections that the colonic structure was intact in the control group and severely disrupted in the DSS group, with destruction of the crypt and disappearance of glands, more severe disruption of the intestinal structure, and intestinal inflammation. The Arec group showed more aggregation of neutrophils than the other two groups (**Figures 2A**). The results of ELISA showed that the levels of inflammatory factors IL-6 (P<0.001), TNF-α (P<0.001), and IL-1β (P<0.001) were significantly higher in the colonic tissue of the Arec group than the DSS group (**Figures 2B–D**).

### Effect of arecoline on intestinal microbiota

**Figure 3:**
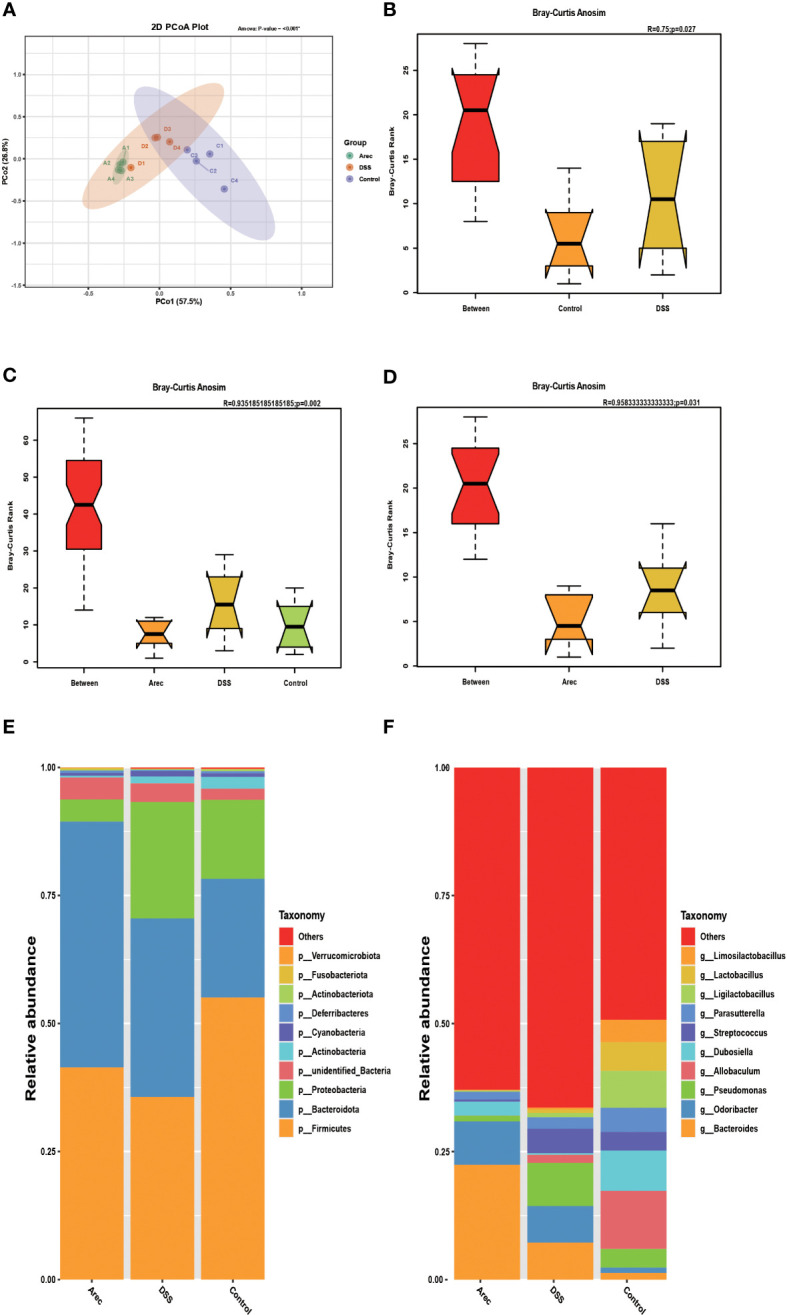
Arecoline and DSS interventions affect changes in intestinal flora. **(A)** PCoA analysis of OTU-based weighted UniFrac distances showed microbial differences between the groups. **(B–D)** OTU-based ANOSIM inter-group variation analysis. **(E)** Gut microbial composition of the three groups of mice at the portal level. **(F)** Intestinal microbial composition of the three groups of mice at the genus level. P<0.05 was statistically significant.

It has been shown that the intestinal microbiota is related to digestion, absorption, metabolism, immunity, and other functions, and that dysbiosis of the intestinal flora is closely related to the occurrence of many diseases such as tumors, diarrhea, obesity, and cardiovascular and cerebrovascular diseases (23–25). Disruptions in the balance of the gut microbiota have also been reported to have many links to the development of UC (26, 27). Therefore, to investigate the effects of arecoline and DSS on intestinal flora, we sequenced 16S rRNA in fecal samples from all three groups and analyzed the alpha and beta diversity indices of the mouse intestinal microbiota. The Arec group had increased gut microbiota diversity as compared to the DSS and control groups according to the Shannon, Simpson, and ACE indices. According to the Chao1 index, the DSS group showed increased diversity of gut microbiota and the Arec group showed decreased diversity of gut microbiota compared to the control group (**Table 1**). OTU-based weighted UniFrac distance PCoA analysis showed significant differences in the gut microbiota composition of the three groups of mice (P<0.001) (**Figure 3A**). ANOSIM showed the same results, with significant differences between the control, DSS, and Arec groups (**Figures 3B–D**). At the phylum level, the Arec group showed an increase in the relative abundance of Bacteroidota and Firmicutes and a decrease in the relative abundance of Proteobacteria and Actinobacteria compared to the DSS group. The relative abundance of Proteobacteria and Bacteroidota increased in the DSS group compared to the control group, while the relative abundance of Firmicutes decreased significantly (**Figure 3E**). At the genus level, the relative abundance of *Bacteroides* and *Odoribacter* increased in the Arec group compared to the DSS group, while the abundance of *Parasutterella*, *Allobaculum*, *Ligilactobacillus*, *Lactobacillus*, and *Limosilactobacillus* decreased significantly (**Figure 3F**).

**Table 1:** Alpha diversity parameters assessed by the Shannon, Simpson, Chao1, and ACE indices.

Group	Shannon	Simpson	Chao1	ACE
Arec	6.275	0.945	833.626	834.783
DSS	5.839	0.962	783.965	793.998
Control	5.562	0.947	765.866	782.761

**Figure 4:**
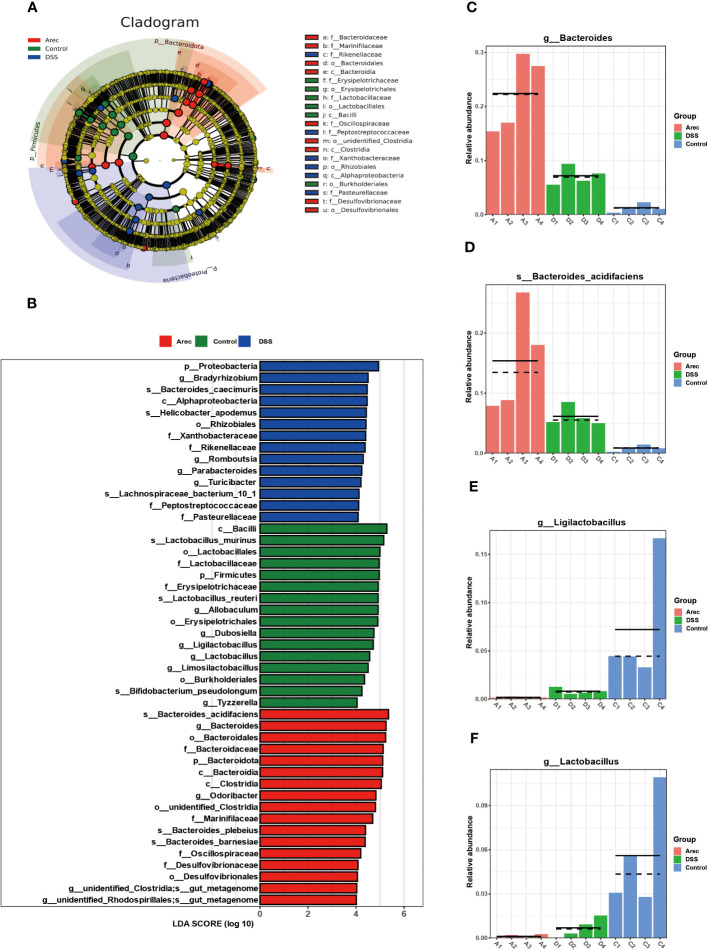
Results of LEfSe analysis of gut microbiota in the Arec, DSS, and control groups. **(A)** Taxonomic cladogram showing the main constituent taxa of the three groups of mouse intestinal microorganisms. **(B)** The distribution of LDA values in the histogram shows the species with LDA score greater than 4. **(C–F)** Difference maps of representative intestinal microorganisms three groups of mice.

In the analysis of LEfSe results, the taxonomic phylogram showed that the main composition of the intestinal microbiota in the three groups of mice was Bacteroidota, Firmicutes, and Proteobacteria (**Figure 4A**). The LDA scores for the abundance of taxonomic units indicated that *Bacteroides* in the Arec group had higher LDA scores than in the DSS and control groups. By contrast, the control group was dominated by *Lactobacillus* (**Figure 4B**). As shown in **Figures 4C–F**, the population of *Bacteroides* and *Bacteroides acidifaciens* was significantly increased in the Arec group compared to the other two groups, while the population of *Ligilactobacillus* and *Lactobacillus* almost disappeared. Therefore, elevated abundance of *Bacteroides* and reduced abundance of *Lactobacillus* may be the reason for arecoline treatment leading to more severe colonic inflammation.

### Effect of arecoline on the metabolism of intestinal flora

**Figure 5:**
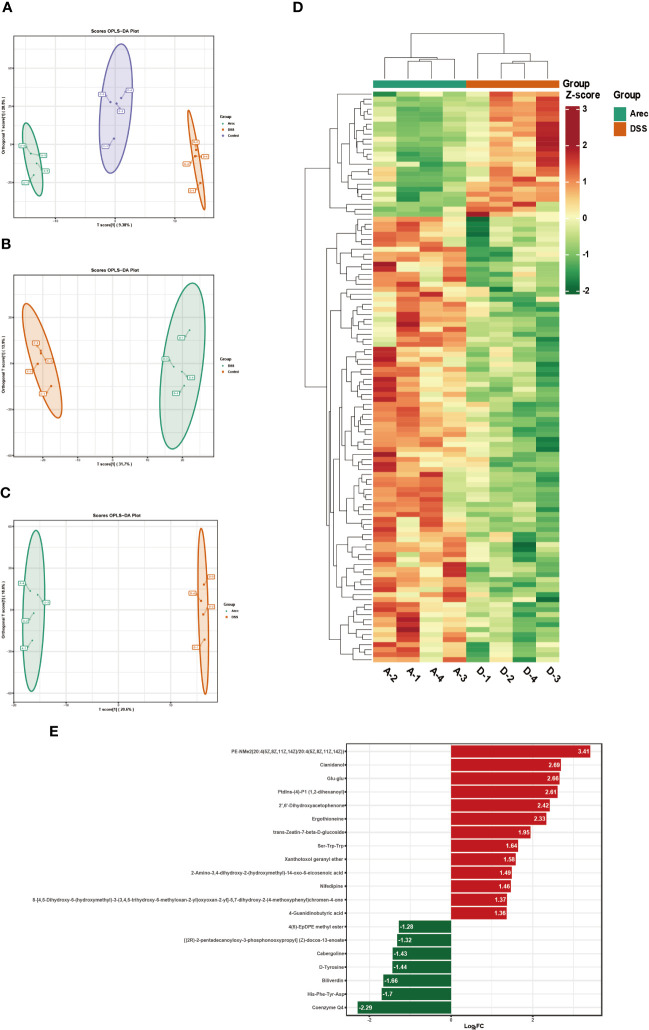
Arecoline regulates serum metabolism in positive-ion mode. **(A–C)** OPLS-DA based analysis showing differences among the Arec, DSS, and control groups; differences between the DSS and control groups, and differences between the Arec and DSS groups. **(D)** Heat map reflects the differential metabolites between the Arec and DSS groups (high levels in red, low levels in green). **(E)** KEGG enrichment maps of differential metabolites in the Arec and DSS groups.

The metabolites of the intestinal microbiota regulate all bodily functions. We analyzed the differential metabolites of the three groups of mice sera by performing untargeted metabolomics assays on their sera. In the positive ion mode, OPLS-DA showed a significant separation of metabolites in the three groups (**Figure 5A**). In addition, the metabolites were significantly separated between the DSS and control groups (**Figure 5B**) and between the Arec and DSS groups (**Figure 5C**). As shown in **Figure 5D**, the clustering analysis of different metabolites between the Arec group and DSS group in positive-ion mode showed that there were 114 differential metabolites between the two groups, with 25 up-regulated and 89 down-regulated ones. As shown in **Figure 5E**, the top 20 metabolic pathways including Nicotinate and nicotinamide metabolism, Metabolic pathways, and Glyoxylate and dicarboxylate metabolism were obtained using KEGG pathway enrichment analysis based on the different metabolite results between the Arec and DSS groups.

**Figure 6:**
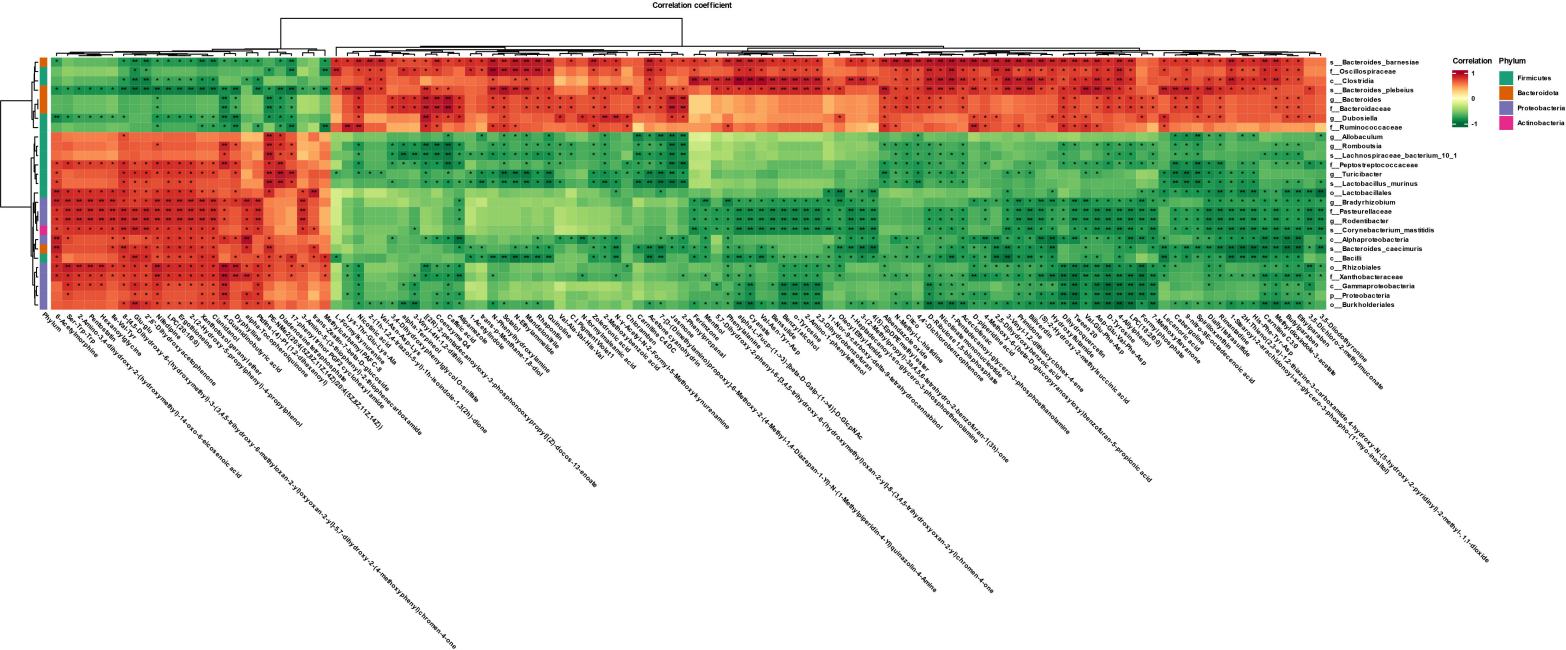
Heat map showing gut microbes associated with differential metabolites in the Arec and DSS groups (red indicates high levels, green indicates low levels). *P<0.05 and **P<0.01.

We performed a joint analysis for intestinal flora and differential metabolites. Pentostatin, nifedipine, LPC (20:1/0:0), ergothioneine, and other metabolites increased with increasing abundance of Proteobacteria and Firmicutes and decreased with increasing abundance of Bacteroidota and Actinobacteria (**Figure 6**).

## Discussion

Ulcerative colitis is a chronic idiopathic inflammatory disease caused by an imbalance of immune mechanisms and environmental factors, which often manifests clinically as diarrhea, bleeding, and malabsorption, and is a significant threat to people’s physical and mental health (2, 3). The clinical signs and pathological changes in mice in the DSS group after 7 days of induction with 3% DSS indicated the success of our UC model. Our experiments revealed that 5 mg/kg arecoline aggravated the symptoms of colitis induced by DSS, and the Arec group showed higher DAI scores and showed more severe weight loss as well as shortening of the colon than the DSS and control groups. Pathological examination of the intestinal tract of mice showed that the DSS group had reduced crypts, mucosal breakdown, and inflammatory cell infiltration; moreover, the breakdown of the intestinal barrier and inflammatory cell infiltration were more severe with the addition of arecoline.

We also detected a significant increase in pro-inflammatory factors (IL-6, IL-1β, and TNF-α) in the Arec group by ELISA. Among them, the pro-inflammatory effect of IL-6 stems from its ability to prevent T-cell apoptosis, but it also affects the pro-ablative and repair functions of the epithelial barrier (28). TNF-α promotes T-cell proliferation and differentiation and aggravates intestinal inflammation (29). Studies have shown that elevated IL-1β correlates with the severity of acute inflammation and that inhibition of IL-1β expression alleviates the symptoms of colitis (30). These experimental results suggest that arecoline promotes the expression of inflammatory factors and aggravates UC; therefore, we investigated its mechanism of action in more depth.

As the study of UC has become more advanced, a large number of studies have shown that intestinal microorganisms play a central role in the development of UC (31, 32). Hence, we collected fecal samples from mice for 16S rRNA sequencing to examine their intestinal flora composition. The sequencing results showed significant differences in the gut microbial composition of the three groups. At the phylum level, the relative abundance of Proteobacteria was significantly increased after DSS treatment compared to the control group, consistent with previous studies. The study showed that the ratio of Firmicutes to Bacteroides (F/B) was reduced in the gut of UC patients (33), consistent with our experimental results, and the reduction of F/B was more significant with the addition of arecoline, which aggravated the dysbiosis of the intestinal flora caused by DSS.

At the genus level, arecoline exacerbated the effect of DSS on beneficial bacterial genera in the intestinal tract. *Lactobacillus* is a well-known beneficial genus in the intestine and has a protective effect on the gut in colitis (34, 35). The abundance of *Lactobacillus* was significantly reduced in the DSS group relative to the control group, and almost disappeared after the use of arecoline. Furthermore, the abundance of *Limosilactobacillus* (36, 37) and *Ligilactobacillus* (38, 39), which have antimicrobial and immune as well as intestinal barrier-enhancing abilities, were inhibited by DSS. *Limosilactobacillus* has been shown to influence the NF-κB signaling pathway to inhibit inflammation and prevent the development and progression of colitis (40). In addition, *Limosilactobacillus* also increases the induction and production of PD-1^+^ T follicular helper cell-dependent IgA, which alters the gut microbiome and prevents DSS-induced colitis and intestinal ecological dysbiosis (41). These results suggest that arecoline further exacerbates DSS-induced colitis by decreasing the abundance of beneficial bacterial genera in the mouse intestine, thereby exacerbating the development of colitis.

In addition, the relative abundance of *Bacteroides* in the intestine of DSS-treated mice was significantly increased in our assay, which is consistent with previous results of studies regarding the potential role of symbiotic *Bacteroides* in the induction of colonic inflammation in mice (42). It has also been reported that enterotoxigenic *Bacteroides fragilis* (ETBF) in *Bacteroides* increases the permeability of intestinal epithelial cells and disrupts epithelial barrier function by secreting enterotoxins, while ETBF colonization leads to acute or chronic inflammation of the intestine (43, 44). Therefore, based on our experimental results, it can be inferred that an increase in the relative abundance of *Bacteroides* may lead to the development of UC. The abundance of *Odoribacter* significantly increased with the use of DSS in our study, which was contrary to previous studies which reported that *Odoribacter* was more abundant in healthy individuals than in those with colitis (45). However, there are also many studies suggesting that *Odoribacter* may act as a conditional pathogenic agent and that its increased relative abundance may be positively associated with the occurrence of UC (46, 47). These results suggest that arecoline increases the relative abundance of conditionally pathogenic bacteria, thereby exacerbating DSS-induced colitis.

We analyzed the main components of intestinal microbiota of the three groups of mice by LEfSe analysis, in which the probiotic *Lactobacillus* obtained a higher score in the control group and the conditional pathogenic bacteria *Bacteroides* was higher in the Arec group. These results further corroborate our inference.

Intestinal microbiota can regulate immunity, maintain the intestinal environment, and provide energy to the body through its metabolites (48). For example, tryptophan and its metabolites can alleviate intestinal inflammation by reducing pro-inflammatory factors such as IL-22, and IL-17 (49). The production of SCFAs is essential for intestinal integrity, as they regulate intestinal pH and influence intestinal mucus production, thereby increasing mucosal immune function (50). In our study, metabolites such as ergothioneine, pentostatin, LPC (20:1/0:0), Glu-glu, and diadenosine tetraphosphate were reduced after arecoline treatment when compared with the DSS group. Ergothioneine is a common oxidant with strong antioxidant activity. It has been shown that oral administration of ergothioneine can increase the level of anti-inflammatory factors in the body and inhibit the TLR4/MyD88/NF-κB signaling pathway, thus protecting from colonic shortening and intestinal pathological damage in UC (51). Ergothioneine is thought to be a metabolite of the probiotic bacterium *Limosilactobacillus*, and the elevation of ergothioneine was positively correlated with *Limosilactobacillus* abundance (52). This is also consistent with the results of our combined gut microbial and metabolite analysis, wherein the relative population of *Limosilactobacillus* in the Arec group nearly disappeared, and arecoline may have reduced the amount of ergothioneine in mice by reducing the abundance of *Limosilactobacillus*. Pentostatin is a purine antimetabolite commonly used in the treatment of malignancies, which inhibits adenosine deaminase. Studies have shown that pentostatin can affect the release of pro-inflammatory factors and attenuate the effects of IL-10-/colitis (53). Our combined microbial and metabolite report showed that pentostatin levels increased with Proteobacteria and Firmicutes and decreased with Bacteroidota. Therefore, the decrease in pentostatin likely indicates that arecoline treatment increased the abundance of Bacteroidota and decreased the abundance of Proteobacteria. Diadenosine tetraphosphate is a metabolite produced under stressful conditions such as hypoxia and injury, and is widely found in prokaryotic and eukaryotic organisms for its role in regulating immune responses, gene expression repair, and DNA replication and synthesis (54). When DSS induced stress in mice, diadenosine tetraphosphate was secreted. However, the use of arecoline reduced the serum levels of diadenosine tetraphosphate, and according to our combined microbial and metabolite analysis report, it is possible that arecoline reduced the abundance of *Allobaculum* and *Lactobacillus* and increased the abundance of *Bacteroides*, resulting in a decrease of diadenosine tetraphosphate in mice. According to the metabolic pathway enrichment analysis based on the differential metabolite results, arecoline may aggravate UC in mice by modulating gut microbiota to regulate nicotinate and nicotinamide metabolism, metabolic pathways, and glyoxylate and dicarboxylate metabolism, which are important metabolic pathways. In summary, we suggest that arecoline regulates serum metabolite production by directly affecting the abundance of intestinal microbiota.

## Conclusion

Our study shows that arecoline can aggravate the colonic damage of DSS-induced UC and increase the release of inflammatory factors. Based on our gut microbial sequencing results, we can conclude that UC in arecoline-treated aggravated mice may be mediated by modulation of the gut microbiota resulting in a decrease in the abundance of beneficial intestinal genera and an increase in the abundance of conditionally pathogenic bacteria. Moreover, the combined microbial and metabolite analysis showed that gut microbes were significantly associated with differential serum metabolites, and arecoline exacerbated UC in mice by affecting the abundance of intestinal flora that regulated serum metabolite concentrations. However, the exact mechanism of arecoline action needs to be further confirmed by targeted metabolomics. In addition, further studies are needed to investigate the effects of arecoline on patients with inflammatory bowel disease.

## Data availability statement

The original contributions presented in the study are publicly available. This data can be found here: https://www.ncbi.nlm.nih.gov/bioproject/PRJNA977598.

## Ethics statement

The animal study was reviewed and approved by The Institutional Animal Care and Use Committee of Nanchang Royo Biotech Co., Ltd.

## Author contributions

DL and BY supervised the project and designed this study. HZ and TD performed the experiments and organized the manuscript. YC and WY conducted the data analysis. JR revised the manuscript. All authors contributed to the article and approved the submitted version.

## References

[1] Segal JP LeBlanc JF Hart AL. Ulcerative colitis: an update. Clin Med (Lond) (2021) 21(2):135–9. doi: 10.7861/clinmed.2021-0080 PMC8002778 33762374

[2] Ananthakrishnan AN. Epidemiology and risk factors for IBD. Nat Rev Gastroenterol Hepatol (2015) 12(4):205–17. doi: 10.1038/nrgastro.2015.34 25732745

[3] Ungaro R Mehandru S Allen PB Peyrin-Biroulet L Colombel JF. Ulcerative colitis. Lancet (2017) 389(10080):1756–70. doi: 10.1016/S0140-6736(16)32126-2 PMC6487890 27914657

[4] Yashiro M. Ulcerative colitis-associated colorectal cancer. World J Gastroenterol (2014) 20(44):16389–97. doi: 10.3748/wjg.v20.i44.16389 PMC4248182 25469007

[5] Shen ZH Zhu CX Quan YS Yang ZY Wu S Luo WW. Relationship between intestinal microbiota and ulcerative colitis: mechanisms and clinical application of probiotics and fecal microbiota transplantation. World J Gastroenterol (2018) 24(1):5–14. doi: 10.3748/wjg.v24.i1.5 29358877 PMC5757125

[6] Imhann F Vich Vila A Bonder MJ Fu J Gevers D Visschedijk MC. Interplay of host genetics and gut microbiota underlying the onset and clinical presentation of inflammatory bowel disease. Gut (2018) 67(1):108–19. doi: 10.1136/gutjnl-2016-312135 PMC5699972 27802154

[7] Sang LX Chang B Zhang WL Wu XM Li XH Jiang M. Remission induction and maintenance effect of probiotics on ulcerative colitis: a meta-analysis. World J Gastroenterol (2010) 16(15):1908–15. doi: 10.3748/wjg.v16.i15.1908 PMC2856834 20397271

[8] Wu Y Jha R Li A Liu H Zhang Z Zhang C. Probiotics (Lactobacillus plantarum HNU082) supplementation relieves ulcerative colitis by affecting intestinal barrier functions, immunity-related gene expression, gut microbiota, and metabolic pathways in mice. Microbiol Spectr (2022) 10(6):e0165122. doi: 10.1128/spectrum.01651-22 36321893 PMC9769980

[9] Xue L Zhao Y Wang H Li Z Wu T Liu R. The effects of live and pasteurized akkermansia muciniphila on DSS-induced ulcerative colitis, gut microbiota, and metabolomics in mice. Food Funct (2023) 14(10):4632–46. doi: 10.1039/d2fo03493j 37098829

[10] Zhou Z He W Tian H Zhan P Liu J. Thyme (Thymus vulgaris l.) polyphenols ameliorate DSS-induced ulcerative colitis of mice by mitigating intestinal barrier damage, regulating gut microbiota, and suppressing TLR4/NF-κB-NLRP3 inflammasome pathways. Food Funct (2023) 14(2):1113–32. doi: 10.1039/d2fo02523j 36594593

[11] Parada Venegas D de la Fuente MK Landskron G González MJ Quera R Dijkstra G. Short chain fatty acids (SCFAs)-mediated gut epithelial and immune regulation and its relevance for inflammatory bowel diseases. Front Immunol (2019) 10:277. doi: 10.3389/fimmu.2019.00277 30915065 PMC6421268

[12] Rodríguez-Nogales A Algieri F Garrido-Mesa J Vezza T Utrilla MP Chueca N. Differential intestinal anti-inflammatory effects of lactobacillus fermentum and lactobacillus salivarius in DSS mouse colitis: impact on microRNAs expression and microbiota composition. Mol Nutr Food Res (2017) 61(11):1700144. doi: 10.1002/mnfr.201700144 28752563

[13] Bales A Peterson MJ Ojha S Upadhaya K Adhikari B Barrett B. Associations between betel nut (areca catechu) and symptoms of schizophrenia among patients in Nepal: a longitudinal study. Psychiatry Res (2009) 169(3):203–11. doi: 10.1016/j.psychres.2008.06.006 19748131

[14] Calogero AE Kamilaris TC Gomez MT Johnson EO Tartaglia ME Gold PW. The muscarinic cholinergic agonist arecoline stimulates the rat hypothalamic-pituitary-adrenal axis through a centrally-mediated corticotropin-releasing hormone-dependent mechanism. Endocrinology (1989) 125(5):2445–53. doi: 10.1210/endo-125-5-2445 2551652

[15] Hsu HF Tsou TC Chao HR Shy CG Kuo YT Tsai FY. Effects of arecoline on adipogenesis, lipolysis, and glucose uptake of adipocytes-a possible role of betel-quid chewing in metabolic syndrome. Toxicol Appl Pharmacol (2010) 245(3):370–7. doi: 10.1016/j.taap.2010.04.008 20406654

[16] Hosein M Mohiuddin S Fatima N. Association between grading of oral submucous fibrosis with frequency and consumption of areca nut and its derivatives in a wide age group: a multi-centric cross sectional study from Karachi, Pakistan. J Cancer Prev (2015) 20(3):216–22. doi: 10.15430/JCP.2015.20.3.216 PMC4597811 26473161

[17] Prabhu RV Prabhu V Chatra L Shenai P Suvarna N Dandekeri S. Areca nut and its role in oral submucous fibrosis. J Clin Exp Dent (2014) 6(5):e569–75. doi: 10.4317/jced.51318 PMC4312688 25674328

[18] Chang NW Pei RJ Tseng HC Yeh KT Chan HC Lee MR. Co-Treating with arecoline and 4-nitroquinoline 1-oxide to establish a mouse model mimicking oral tumorigenesis. Chem Biol Interact (2010) 183(1):231–7. doi: 10.1016/j.cbi.2009.10.005 19840781

[19] Cheng SJ Ko HH Cheng SL Lee JJ Chen HM Chang HH. Arecoline-stimulated placenta growth factor production in gingival epithelial cells: modulation by curcumin. Oral Dis (2013) 19(5):513–8. doi: 10.1111/odi.12034 23163860

[20] Haque MF Meghji S Khitab U Harris M. Oral submucous fibrosis patients have altered levels of cytokine production. J Oral Pathol Med (2000) 29(3):123–8. doi: 10.1034/j.1600-0714.2000.290304 10738939

[21] Liu YJ Peng W Hu MB Xu M Wu CJ. The pharmacology, toxicology and potential applications of arecoline: a review. Pharm Biol (2016) 54(11):2753–60. doi: 10.3109/13880209.2016.1160251 27046150

[22] Wirtz S Neufert C Weigmann B Neurath MF. Chemically induced mouse models of intestinal inflammation. Nat Protoc (2007) 2(3):541–6. doi: 10.1038/nprot.2007.41 17406617

[23] Yano JM Yu K Donaldson GP Shastri GG Ann P Ma L. Indigenous bacteria from the gut microbiota regulate host serotonin biosynthesis. Cell (2015) 161(2):264–76. doi: 10.1016/j.cell.2015.02.047 PMC4393509 25860609

[24] Reinhardt C Bergentall M Greiner TU Schaffner F Ostergren-Lundén G Petersen LC. Tissue factor and PAR1 promote microbiota-induced intestinal vascular remodelling. Nature (2012) 483(7391):627–31. doi: 10.1038/nature10893 PMC3885420 22407318

[25] Gomaa EZ. Human gut microbiota/microbiome in health and diseases: a review. Antonie Van Leeuwenhoek (2020) 113(12):2019–40. doi: 10.1007/s10482-020-01474-7 33136284

[26] Glassner KL Abraham BP Quigley EMM. The microbiome and inflammatory bowel disease. J Allergy Clin Immunol (2020) 145(1):16–27. doi: 10.1016/j.jaci.2019.11.003 31910984

[27] Ohkusa T Koido S. Intestinal microbiota and ulcerative colitis. J Infect Chemother (2015) 21(11):761–8. doi: 10.1016/j.jiac.2015.07.010 26346678

[28] Friedrich M Pohin M Powrie F. Cytokine networks in the pathophysiology of inflammatory bowel disease. Immunity (2019) 50(4):992–1006. doi: 10.1016/j.immuni.2019.03.017 30995511

[29] Billmeier U Dieterich W Neurath MF Atreya R. Molecular mechanism of action of anti-tumor necrosis factor antibodies in inflammatory bowel diseases. World J Gastroenterol (2016) 22(42):9300–13. doi: 10.3748/wjg.v22.i42.9300 PMC5107694 27895418

[30] Torretta S Scagliola A Ricci L Mainini F Di Marco S Cuccovillo I. D-mannose suppresses macrophage IL-1β production. Nat Commun (2020) 11(1):6343. doi: 10.1038/s41467-020-20164-6 33311467 PMC7733482

[31] Gonzalez CG Mills RH Zhu Q Sauceda C Knight R Dulai PS. Location-specific signatures of crohn’s disease at a multi-omics scale. Microbiome (2022) 10(1):133. doi: 10.1186/s40168-022-01331-x 35999575 PMC9400277

[32] Schirmer M Denson L Vlamakis H Franzosa EA Thomas S Gotman NM. Compositional and temporal changes in the gut microbiome of pediatric ulcerative colitis patients are linked to disease course. Cell Host Microbe (2018) 24(4):600–610.e4. doi: 10.1016/j.chom.2018.09.009 30308161 PMC6277984

[33] Kabeerdoss J Jayakanthan P Pugazhendhi S Ramakrishna BS. Alterations of mucosal microbiota in the colon of patients with inflammatory bowel disease revealed by real time polymerase chain reaction amplification of 16S ribosomal ribonucleic acid. Indian J Med Res (2015) 142(1):23–32. doi: 10.4103/0971-5916.162091 26261163 PMC4557246

[34] Wu J Wei Z Cheng P Qian C Xu F Yang Y. Rhein modulates host purine metabolism in intestine through gut microbiota and ameliorates experimental colitis. Theranostics (2020) 10(23):10665–79. doi: 10.7150/thno.43528 PMC7482825 32929373

[35] Matos RC Leulier F. Lactobacilli-host mutualism: “learning on the fly”. Microb Cell Fact. (2014) 13 Suppl 1(Suppl 1):S6. doi: 10.1186/1475-2859-13-S1-S6 25186369 PMC4155823

[36] Walter J Britton RA Roos S. Host-microbial symbiosis in the vertebrate gastrointestinal tract and the lactobacillus reuteri paradigm. Proc Natl Acad Sci U S A. (2011) 108 Suppl 1(Suppl 1):4645–52. doi: 10.1073/pnas.1000099107 PMC3063604 20615995

[37] Piccioni A Franza L Vaccaro V Saviano A Zanza C Candelli M. Microbiota and probiotics: the role of limosilactobacillus reuteri in diverticulitis. Medicina (Kaunas) (2021) 57(8):802. doi: 10.3390/medicina57080802 34441008 PMC8398895

[38] Guerrero Sanchez M Passot S Campoy S Olivares M Fonseca F. Ligilactobacillus salivarius functionalities, applications, and manufacturing challenges. Appl Microbiol Biotechnol (2022) 106(1):57–80. doi: 10.1007/s00253-021-11694-0 34889985

[39] Yakabe K Higashi S Akiyama M Mori H Murakami T Toyoda A. Dietary-protein sources modulate host susceptibility to clostridioides difficile infection through the gut microbiota. Cell Rep (2022) 40(11):111332. doi: 10.1016/j.celrep.2022.111332 36103838

[40] Liu B Yang L Wu Y Zhao X. Protective effect of *Limosilactobacillus fermentum* HFY06 on dextran sulfate sodium-induced colitis in mice. Front Microbiol (2022) 13:935792. doi: 10.3389/fmicb.2022.935792 36171753 PMC9512270

[41] Liu HY Giraud A Seignez C Ahl D Guo F Sedin J. Distinct b cell subsets in peyer’s patches convey probiotic effects by limosilactobacillus reuteri. Microbiome (2021) 9(1):198. doi: 10.1186/s40168-021-01128-4 34602091 PMC8487498

[42] Bloom SM Bijanki VN Nava GM Sun L Malvin NP Donermeyer DL. Commensal bacteroides species induce colitis in host-genotype-specific fashion in a mouse model of inflammatory bowel disease. Cell Host Microbe (2011) 9(5):390–403. doi: 10.1016/j.chom.2011.04.009 21575910 PMC3241010

[43] Zamani S Hesam Shariati S Zali MR Asadzadeh Aghdaei H Sarabi Asiabar A Bokaie S. Detection of enterotoxigenic *Bacteroides fragilis* in patients with ulcerative colitis. Gut Pathog (2017) 9:53. doi: 10.1186/s13099-017-0202-0 28924454 PMC5599888

[44] Lucke K Miehlke S Jacobs E Schuppler M. Prevalence of bacteroides and prevotella spp. ulcerative colitis J Med Microbiol (2006) 55(Pt 5):617–24. doi: 10.1099/jmm.0.46198-0 16585651

[45] Zhou W Sailani MR Contrepois K Zhou Y Ahadi S Leopold SR. Longitudinal multi-omics of host-microbe dynamics in prediabetes. Nature (2019) 569(7758):663–71. doi: 10.1038/s41586-019-1236-x PMC6666404 31142858

[46] Wei D Xie L Zhuang Z Zhao N Huang B Tang Y. Gut microbiota: a new strategy to study the mechanism of electroacupuncture and moxibustion in treating ulcerative colitis. Evid Based Complement Alternat Med (2019) 2019:9730176. doi: 10.1155/2019/9730176 31354859 PMC6632505

[47] Li W Sun Y Dai L Chen H Yi B Niu J. Ecological and network analyses identify four microbial species with potential significance for the diagnosis/treatment of ulcerative colitis (UC). BMC Microbiol (2021) 21(1):138. doi: 10.1186/s12866-021-02201-6 33947329 PMC8097971

[48] Lee WJ Hase K. Gut microbiota-generated metabolites in animal health and disease. Nat Chem Biol (2014) 10(6):416–24. doi: 10.1038/nchembio.1535 24838170

[49] Sun M Ma N He T Johnston LJ Ma X. Tryptophan (Trp) modulates gut homeostasis *via* aryl hydrocarbon receptor (AhR). Crit Rev Food Sci Nutr (2020) 60(10):1760–8. doi: 10.1080/10408398.2019.1598334 30924357

[50] Blaak EE Canfora EE Theis S Frost G Groen AK Mithieux G. Short chain fatty acids in human gut and metabolic health. Benef Microbes (2020) 11(5):411–55. doi: 10.3920/BM2020.0057 32865024

[51] Pang L Wang T Liao Q Cheng Y Wang D Li J. Protective role of ergothioneine isolated from pleurotus ostreatus against dextran sulfate sodium-induced ulcerative colitis in rat model. J Food Sci (2022) 87(1):415–26. doi: 10.1111/1750-3841.15982 34873706

[52] Matsuda Y Ozawa N Shinozaki T Wakabayashi KI Suzuki K Kawano Y. Ergothioneine, a metabolite of the gut bacterium lactobacillus reuteri, protects against stress-induced sleep disturbances. Transl Psychiatry (2020) 10(1):170. doi: 10.1038/s41398-020-0855-1 32467627 PMC7256047

[53] Brown JB Lee G Grimm GR Barrett TA. Therapeutic benefit of pentostatin in severe IL-10-/- colitis. Inflammation Bowel Dis (2008) 14(7):880–7. doi: 10.1002/ibd.20410 PMC3065943 18340641

[54] Ferguson F McLennan AG Urbaniak MD Jones NJ Copeland NA. Re-evaluation of diadenosine tetraphosphate (Ap4A) from a stress metabolite to *Bona fide* secondary messenger. Front Mol Biosci (2020) 7:606807. doi: 10.3389/fmolb.2020.606807 33282915 PMC7705103

